# Estimating the effect of hormonal contraceptive use on anemia: A cross-sectional comparative analysis of 46 Demographic and Health Surveys

**DOI:** 10.1371/journal.pone.0327083

**Published:** 2025-07-16

**Authors:** Stephanie R. Chung, Martina X. Spain, Emily Hoppes, Amelia Mackenzie

**Affiliations:** 1 Contraceptive Research, Development, and Introduction. Global Health and Population, FHI 360. Durham, United States of America; 2 Department of Maternal and Child Health, Gillings School of Global Public Health, University of North Carolina at Chapel Hill, Chapel Hill, United States of America; Kasr Alainy Medical School, Cairo University, EGYPT

## Abstract

**Background:**

Anemia is a significant health concern for women in low- and middle-income countries. Hormonal contraceptives, which can reduce heavy menstrual bleeding and prevent pregnancy and its resulting blood loss, are an underexplored opportunity for anemia reduction.

**Methods:**

Using Demographic and Health Survey data from 46 countries across four global regions, we examined country prevalence of anemia (i.e., hemoglobin below 12 g/dl via HemoCue testing) among women aged 15–49 years who were not pregnant. For each country, we used multivariable logistic regression to compare odds of anemia between women using hormonal contraceptive (users) to women using non-hormonal contraception or not using any contraception (nonusers). We merged data across countries to examine this effect stratified by contraceptive method and length of use and conducted a quantitative bias analysis of the anemia test.

**Results:**

Anemia prevalence ranged from 12% in Rwanda to 63% in Gabon. In the merged sample, the crude odds ratio comparing anemia in users of hormonal contraception to nonusers was 0.57 (95% confidence interval: 0.56–0.58); the adjusted odds ratio was 0.55 (0.39–0.79) when controlling for age, education, wealth, and rurality. In the stratified analysis of merged data, we found that users of all hormonal contraception had lower adjusted odds of anemia compared to nonusers (AOR: 0.51, 95% CI: 0.46–0.57), and that the protective effect was strongest among users of contraceptive injections (AOR:0.37, 95% CI 0.30–0.45). In our bias analysis, the crude odds of anemia for users ranged from 0.29 to 0.57 when varying anemia test sensitivity and specificity between 75% and 100%.

**Conclusions:**

Future research should directly examine the causal link between hormonal contraceptive use and anemia as a high impact non-contraceptive benefit of hormonal contraception, especially with understudied methods such as injectable contraception and contraceptive implants.

## Introduction

Anemia is a significant global health concern that impacts quality of life, economic productivity, and mental and physical development for an estimated 1.92 billion people worldwide [1]. Symptoms can include weakness, fatigue, and difficulty concentrating, and when severe, anemia can increase the risk of poor birth outcomes such as preterm labor, low birth weight, and child and infant mortality [2,3]. Iron deficiency anemia is the most common form, accounting for over 60% of the global disease burden [2]. Iron deficiency anemia can stem from multiple causes, including blood loss related to menstruation or pregnancy, as well as diet, and blood loss due to infection by parasites [2,4]. Women of reproductive age are, therefore, at high risk of anemia and often bear the majority of the health and economic costs compared to men [2]. The negative effects of heavy menstrual bleeding on women has been well documented; in addition to many other impacts, heavy menstrual bleeding can increase risk of anemia [5,6]. Preventing and treating anemia in women of reproductive age can greatly improve women’s health and quality of life while also having important population health implications. Preconception and pregnancy are times of heightened concern around anemia; when the birthing parent has low iron stores, infants may have an iron deficiency that could impact their physical and mental development [3]. Despite the global importance attributed to anemia, including the Sustainable Development Goal Indicator 2.2.3 of reducing anemia by half in women and children between 2012 and 2030, there has been little global progress toward this goal [7,8]. Therefore, it is of public health importance to research strategies and interventions to address anemia in women of reproductive age, especially in low- and middle-income countries (LMICs) where the prevalence of anemia is notably higher compared to high-income countries [2].

Family planning methods allow for delaying or avoiding pregnancy, lengthening the time between pregnancies, and—for hormonal contraceptive methods like oral contraceptive pills, injectable contraception, contraceptive implants, and hormonal intrauterine devices (IUD)—reducing or entirely pausing menstrual blood loss [9]. In general, the estrogens and progestins present in hormonal contraceptives thin the endometrial lining of the uterus which often leads to lighter bleeding or amenorrhea, which can affect anemia status [10,11]. The hormonal IUD does this primarily through local progestin release, while the others act systemically [12]. These bleeding changes caused by hormonal contraception are examples of a wider constellation of ways contraception impacts the menstrual cycle, referred to as contraceptive-induced menstrual changes (CIMCs), which can be a key factor in contraceptive decision-making [13]. These impacts are highly personal and context-specific; previous research has found a range of positive and negative responses to CIMCs, with lighter bleeding generally viewed positively, but with both positive and negative responses to completely paused bleeding [13,14].

Given hormonal contraception can reduce menstrual blood loss, there is a theoretical non-contraceptive benefit of use to prevent or treat anemia. However, different bleeding profiles across different methods of hormonal contraception mean some contraceptive methods may be more protective against anemia than others. For example, most combined oral contraceptives include placebo pills for 4 or 7 days to induce withdrawal bleeding designed to mimic menstruation, though this bleeding is often less than a typical menstrual period. Bleeding can be further reduced by continuous use of combined oral contraception, i.e., not taking the placebo pills, and several marketed formulations provide extended, 3-month coverage between withdrawal bleeding; however, it is not evident that these formulations and the continuous-use practice are common in LMICs. Additionally, oral contraceptive pills require high user adherence to daily pill taking, and most women do not achieve 100% adherence, which can result in breakthrough bleeding or pregnancy [15,16]. Still, small-scale studies in high-income countries have shown associations between oral contraceptive pill use, reduction in menstrual blood loss, and subsequent reductions in anemia and iron deficiency [17,18].

In addition to oral contraceptive pills, the contraceptive implant or injectable contraception can reduce or pause bleeding [19,20]. Compared to oral contraceptive pills, however, these longer acting methods require less user involvement, such as remembering to take a daily pill [21,22]. Another long-acting method, the hormonal IUD, has the potential to manage many menstrual and gynecologic disorders and symptoms including heavy menstrual bleeding, and there is currently a clinical trial [NCT05233956] to investigate its impact on anemia [23]. Although there are current efforts to increase access to the hormonal IUD in LMICs due to its high effectiveness and non-contraceptive benefits, its use has historically been very limited. The non-hormonal IUD, on the other hand, has been shown to increase bleeding, which could enhance the risk of anemia [24–27]. Determining whether, and to what extent, various types of hormonal contraceptives have the potential for anemia treatment or prevention may provide important information for those using or considering hormonal contraception, potentially moving the needle on a condition that has been challenging to address.

There is a growing body of research in LMICs exploring the relationship between hormonal contraception and anemia [28–30]. In 2012, Bellizzi and Ali found an association between oral contraceptive use and lower odds of having anemia (OR 0.68, 95% CI: 0.64–0.73) using Demographic and Health Surveys (DHS) data from in 12 LMICs, with the effect increasing with longer duration of pill use [29]. More recently, using DHS data from 51 LMICs, Misunas et al. found use of the contraceptive pill, injectable contraception, or contraceptive implant significantly reduced the odds of mild, moderate, and severe anemia in adolescent girls and young women aged 15–24 [28], and Aboagye et al. found similar results among all women of reproductive age in DHS from 14 sub-Saharan African countries [31]. Using DHS data from Ethiopia, Tshome et al. found users of modern contraceptive methods, which includes hormonal contraceptives, had lower odds of anemia than women using barrier methods of contraception, such as condoms [30]. Although most of the research on LMICs come from analysis of DHS data, there have been smaller community-based studies in LMICs that have also found associations between hormonal contraceptive use and anemia reduction [32,33]. Overall, this research suggests there is an association between hormonal contraceptive use and anemia reduction in LMICs, but it has largely focused on either solely oral contraceptive pill use or hormonal IUD use, or it has conflated hormonal contraceptive methods together. Additionally, there has been concern that the HemoCue testing system used to determine anemia status in DHS surveys may over- or under-estimate true anemia rates, introducing measurement error into analyses [34–39]. There is a need for further comparative studies to understand to what extent hormonal contraceptives, and specific hormonal methods, can be protective at a population level against anemia, and to what extent population level estimates of anemia may be biased.

Our study seeks to address several previous gaps in the literature. Specifically, previous studies have limited analysis to one method of contraception, or investigated multiple methods only in specific demographic populations, countries, or regions [24–26,28–32,40]. Additionally, few previous studies have conducted robust sensitivity analyses, explored moderation effects by method type, or addressed concerns about measurement error in anemia testing systems. Our analysis builds upon previous research by further exploring the relationship between hormonal contraceptive use and anemia, in aggregate and by individual method, over multiple durations of use for women of reproductive age in 46 countries across four global regions and by assessing the robustness of our results when accounting for measurement error in anemia point-of-care testing systems.

## Methods

### Sample

We used data from 46 publicly available DHS. Data were included if the DHS was the most recent from that country conducted between the years 2008–2023, included anemia testing in their women’s questionnaire, and were missing less than 10% of anemia testing results (n.b., because DHS use the term “women”, we use the same terminology, although not everyone who uses hormonal contraception, menstruates, or can become pregnant identifies as a woman). We reviewed all 64 DHS from 2008–2023 that were available in August 2023, and 46 met our inclusion criteria. The 29 sub-Saharan African surveys included Benin (2017/18), Burkina Faso (2010), Burundi (2016), Cameroon (2018), Congo (2011), Cote d’Ivoire (2011/12), Democratic Republic of the Congo (2013/14), Ethiopia (2016), Gabon (2019/21), Gambia (2019/20), Ghana (2014), Guinea (2019), Lesotho (2014), Liberia (2019/20), Madagascar (2021), Malawi (2015/16), Mali (2018), Mauritania (2019/21), Mozambique (2011), Namibia (2013), Niger (2012), Nigeria (2018), Rwanda (2019/20), Sao Tome and Principe (2008/09), Tanzania (2015/16), Togo (2013/14), Uganda (2016), Zambia (2018), and Zimbabwe (2015); the 6 North Africa/West Asia/Eastern Europe surveys included Albania (2017/18), Armenia (2015/16), Egypt (2014), Jordan (2017/18), Kyrgyz Republic (2012), and Tajikistan (2017); the 5 South/Southeast Asia surveys included Cambodia (2014), India (2019/21), Myanmar (2015/16), Nepal (2022), and Timor Leste (2016); and the 6 Latin America and Caribbean surveys included Bolivia (2008), Guatemala (2014), Guyana (2009), Haiti (2016/17), Honduras (2011/12), and Peru (2012). Of the 64 DHS we reviewed, 14 did not report anemia and four (Maldives, Sierra Leone, South Africa, and Yemen) were missing more than 10% of anemia results. The merged dataset for all 46 DHS included 1,375,291 women.

All data was de-identified before it was shared by DHS, and authors had no access to information that could be used to identify participants after data collection. The DHS program obtains ethical approval from the ICF Institutional Review Board (IRB) and country-specific IRBs in the host countries. Before the survey is administered and biomarker tests are conducted, an informed consent protocol is used to ethically enroll participants. More information can be found at the DHS website [41].

### Complex sampling design

DHS use complex sampling designs to conduct population-based household surveys yielding representative national and subnational estimates. For each DHS, a probability sample of households are typically selected using a two-stage cluster design generally using census enumeration areas from an existing national sampling frame, with country-specific stratification, usually subnational region and urban/rural strata. In each selected household, all women of reproductive age (typically 15–49) who are *de jure* residents of the household are eligible for the women’s questionnaire. The details of the sampling design used in each country can be found in their respective DHS reports [42]. For our outcome of interest, anemia, some DHS only conduct anemia testing for women from a probability sample of the selected households instead of for all women as part of the women’s questionnaire.

### Measures

#### Exposure.

Data on the exposure, hormonal contraceptive use, are collected in the woman’s questionnaire. Women are asked if they or their partner are currently doing something to prevent pregnancy. If yes, they are asked the method(s) they are using. We defined hormonal contraceptive users as woman who reported using—singly or in combination with another method—oral contraceptive pills, the contraceptive implant, or injectable contraception. The DHS does not distinguish between hormonal IUDs, which can cause less menstrual bleeding, and non-hormonal IUDs, which can cause increased menstrual bleeding; therefore, we did not include IUD users (n = 23,694) in the analysis sample. Women reporting female sterilization (n = 196,506) were also not included, as sterilization can have varied effects on the menstrual cycle [43]. We excluded hormonal contraceptive users if they had not been using their method for at least 6 months (n = 20,182) to ensure the method had enough time to impact anemia and the contraceptive-induced menstrual changes had time to stabilize. We also excluded women who were over 49 years old (n = 4,801), as only some DHS include women above this age. All other women were considered non-users of hormonal contraception.

Therefore, our exposed group (**users of hormonal contraception**) included women using oral contraceptive pills, the contraceptive implant, or injectable contraception for more than 6 months, and our reference group (**nonusers of hormonal contraception**) included all women who did not use any form of contraception or women using non-hormonal forms of contraception, including male and female condoms, male sterilization, periodic abstinence, the standard days method, withdrawal, lactational amenorrhea, spermicides, or traditional/folk methods, as defined by the DHS.

#### Outcome.

DHS measure anemia status using 1–3 drops of capillary blood from a finger prick sample using the HemoCue 201+ or 301 + system, which provides a reading of blood hemoglobin concentration in grams per deciliter (g/dL) in one minute [44,45]. The HemoCue system is intended to be a screening, not a diagnostic, tool for anemia, and can only screen for general anemia, not specifically iron deficiency anemia, as it only measures blood hemoglobin and not and individual’s iron reserves. Because anemia is defined differently for women who are pregnant due to changes in blood volume and other factors, we did not include pregnant women (n = 56,719) in our analysis sample as part of the referent non-user group. DHS categorizes non-pregnant women as having anemia if their hemoglobin is below 12 g/dL, with mild anemia between 11.0–11.9 g/dL, moderate anemia between 8.0–10.9 g/dL, and severe anemia below 8.0 g/dL [46]. We dichotomized the outcome as either having anemia (1) or not having anemia (0), collapsing the categories of mild, moderate, and severe anemia.

#### Covariates.

We used a directed acyclic graph (DAG) [47,48] to identify an adjustment set of covariates, including age, wealth, smoking, education, and location (S1 Fig). DAGs are commonly used in epidemiological research to graphically depict causal relationships between variables; to create our DAG, we used the results of a thorough literature review on anemia and links between anemia and hormonal contraceptive use and our subject matter expertise [49]. Using the DAG, we identified causal and confounding pathways between our exposure of interest (hormonal contraceptive use) and our outcome (anemia), then identified which variables we need to adjust for in order to control for confounding pathways. Age was measured continuously by year, and we used a seven-level categorical variable of five-year age groups (i.e., 15–19, 20–24, etc.) for analysis, although we explored four other functional forms, including linear, quadratic, categorical, and quadratic splines. LOESS plots and precision for each functional form were similar, so we chose the categorical variable for ease of comparison with other similar studies. Wealth, used in this analysis as a proxy for social inequality, was measured as a five-level categorical variable using the DHS wealth index which uses an equation that creates wealth quintiles by considering dwelling and household characteristics, access to consumer goods and services, assets, health outcomes and the Gini coefficient, which indicates the level of wealth inequality in that country [50]. Smoking was a binary variable, measured as a self-report of tobacco cigarette consumption. Education was measured by asking women their highest level of completed education, and this was recoded as a 4-level categorical variable for completed schooling, including no school, primary school, secondary school, or more than secondary school to permit comparison across countries. Rural or urban status of residence (i.e., rurality) was determined for each country then verified during data collection and reported as a binary variable.

#### Models.

**Fig 1:**
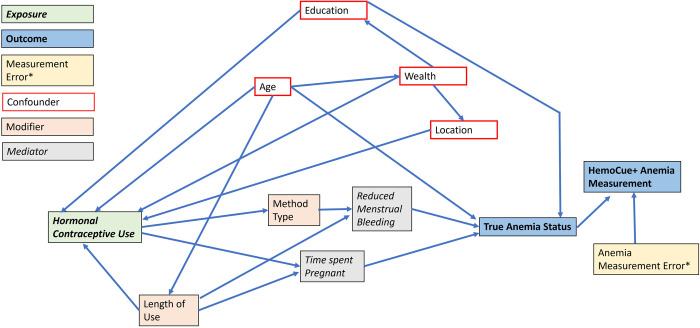
Simplified directed acyclic graph (DAG) illustrating relationship between hormonal contraceptive use and anemia status, with final covariate set. For a full DAG with all variables considered, please see Supplementary S1 Fig.

We used our DAG to determine which covariates we would control for in our models to study the relationship between hormonal contraceptive use and anemia. Based only on the DAG, we would control for age, wealth, rurality, smoking, and education. Based on our knowledge of the literature and expert recommendation, we tested an interaction term for rurality and found no evidence of effect modification (likelihood ratio test statistic: 2.3, p = 0.13), and therefore left this interaction term out of the final model. We weighed the benefits and drawbacks of retaining smoking and educational attainment as confounders. Removing smoking resulted in no change to the estimate or confidence intervals, likely due to the very low prevalence of smoking in our sample (0.8%) and the fact that the DHS corrects anemia levels for smoking and altitude. Therefore, we did not include smoking as a confounder and were able to increase our sample size by 10,372. There was a small change in estimates when removing educational attainment, therefore, we retained it in our model. As shown in Fig 1, our final model controlled for age, wealth, education, and rurality. A more detailed DAG, which takes into account potential confounders that are unmeasured in the DHS (including infections, dietary intake, and underlying health conditions), can be found in S1 Fig.

### Regression analysis of country-level data

We used logistic regression for each included country DHS, comparing users of hormonal contraception to nonusers of hormonal contraception. All analyses were done in Stata 17. We used the provided DHS sampling weights, Stata svy commands and sampling variables provided by DHS to account for the complex sampling design, and the Stata subpop option to appropriately restrict our analysis sample in order to avoid underestimating standard errors. In DHS where only women from a sample of households are tested for anemia, we used the provided household selection variable as a secondary sampling unit within the svy code. For all analyses, we present unweighted sample sizes and weighted percentages.

### Moderation analysis of merged dataset

To identify if method type or length of hormonal contraceptive method use were moderating the relationship between hormonal contraceptive use and anemia, we merged the DHS datasets from all countries and stratified the results by method type and length of use. Again, we used the provided DHS sampling weights and variables, and the Stata svy and subpop commands in this analysis, but we used a country variable as our primary sampling unit in this merged analysis. We hypothesized that the strength of the relationship would be greater for: (a) contraceptive implants and injectable contraception than for oral contraceptive pills, as those longer-acting methods require less user action and are more likely to reduce or entirely stop menstrual bleeding, and (b) for longer lengths of hormonal contraceptive use. We compared the strength of the association for contraceptive implants, injectable contraception, or oral contraceptive pills and for any duration, 6–12 months of use, 13–24 months of use, and more than 25 months of use, overall and by method. For these analyses only, we did not exclude those who had been using hormonal contraception for less than 6 months.

### Analysis of outcome misclassification

Much previous research into anemia has relied heavily on HemoCue testing for determination of anemia status, despite concerns raised by experts on the accuracy of this testing system [34,37,39]. There is evidence the HemoCue testing system used by DHS can either overestimate or underestimate the concentration of hemoglobin in the blood in validation studies, and that accuracy can be impacted by external factors, such as temperature and humidity [34–39]. Compared to the gold standard of a Sysmex autoanalyzer with venous blood, the HemoCue 201+ or 301 + systems can have variable test sensitivity (i.e., ability to detect true positives while avoiding false negatives) and variable test specificity (i.e., ability to detect true negatives while avoiding false positives). The HemoCue sensitivity and specificity can also range depending on the skill level of the person administering the test, with one recent study finding sensitivity ranged from 79% to 94% and specificity ranged from 91% to 100% [51]. Still, other studies have found conflicting results, where the HemoCue systems showed high sensitivity and low specificity, for example, one study in India among pregnant women that found the HemoCue 201+ and 301 + had sensitivity of 93% and 90%, respectively, and specificity of 76% and 80%, respectively [52].

Given these concerns about testing accuracy, we wanted to assess if our results were robust to misclassification of anemia status due to the HemoCue testing system. We varied both the sensitivity and specificity of the HemoCue testing between 75% to 100% to see how the unweighted crude odds of anemia in the merged dataset would be impacted. We used the unweighted crude data rather than presenting more complex probabilistic or multiple bias analyses, although we suggest future work explore these relationships. For this analysis, there is no theoretical reason to expect this misclassification bias would be differential by exposure status (i.e., that any misclassification of anemia by HemoCue testing would be more or less substantial among users of hormonal contraceptives compared to nonusers).

### Sensitivity analyses

We conducted multiple sensitivity analyses in our merged sample around inclusion into our exposed (i.e., users of hormonal contraception) vs. our reference group (i.e., nonusers of hormonal contraception) and the impact of specific countries on our merged analysis. In creating our exposed and reference groups, we excluded those using IUDs and those using female sterilization. We conducted sensitivity analyses where we included those using IUDs in the reference group (assuming that copper IUDs were more likely to be used than hormonal IUDs in these data) and including users of female sterilization in the reference group. We found no substantial differences (i.e., no change in estimates greater than 0.02 in our general model or the moderation analysis), so we continued with excluding IUD users and users of female sterilization from the sample. Despite seeing no difference in estimate, excluding these users is a more conservative approach that prevents exposure misclassification bias. We also conducted a sensitivity analysis where we excluded those women in the reference group who had discontinued a hormonal method in the last six months, in case there was any lingering effect of this previous hormonal contraceptive use. This also had no notable impact on our results (i.e., no change in estimates greater than 0.02 in our general model and the moderation analyses), so we retained these women in the reference group, again to maintain the more conservative approach. Finally, we conducted multiple sensitivity analyses for the moderation analysis, such as including or excluding countries with large sample sizes, like India and Nigeria, and countries with high rates of anemia like India and Gabon (see more detail in Supplementary Material S1 Appendix). The result of this sensitivity analysis changed how we created our sample for the moderation analysis. First, we found that including the 2019–2021 India DHS substantially increased anemia prevalence in our merged dataset. Then, we found a unique moderation effect by method type in the India DHS that was unseen in other countries. We determined that this moderation effect unduly biased the outcomes of our moderation analysis, so we excluded India from the moderation analysis. For additional detail on the reason for this exclusion and an exploration of the moderation analysis with all countries included, see supplementary material.

## Results

### Analysis sample

**Fig 2:**
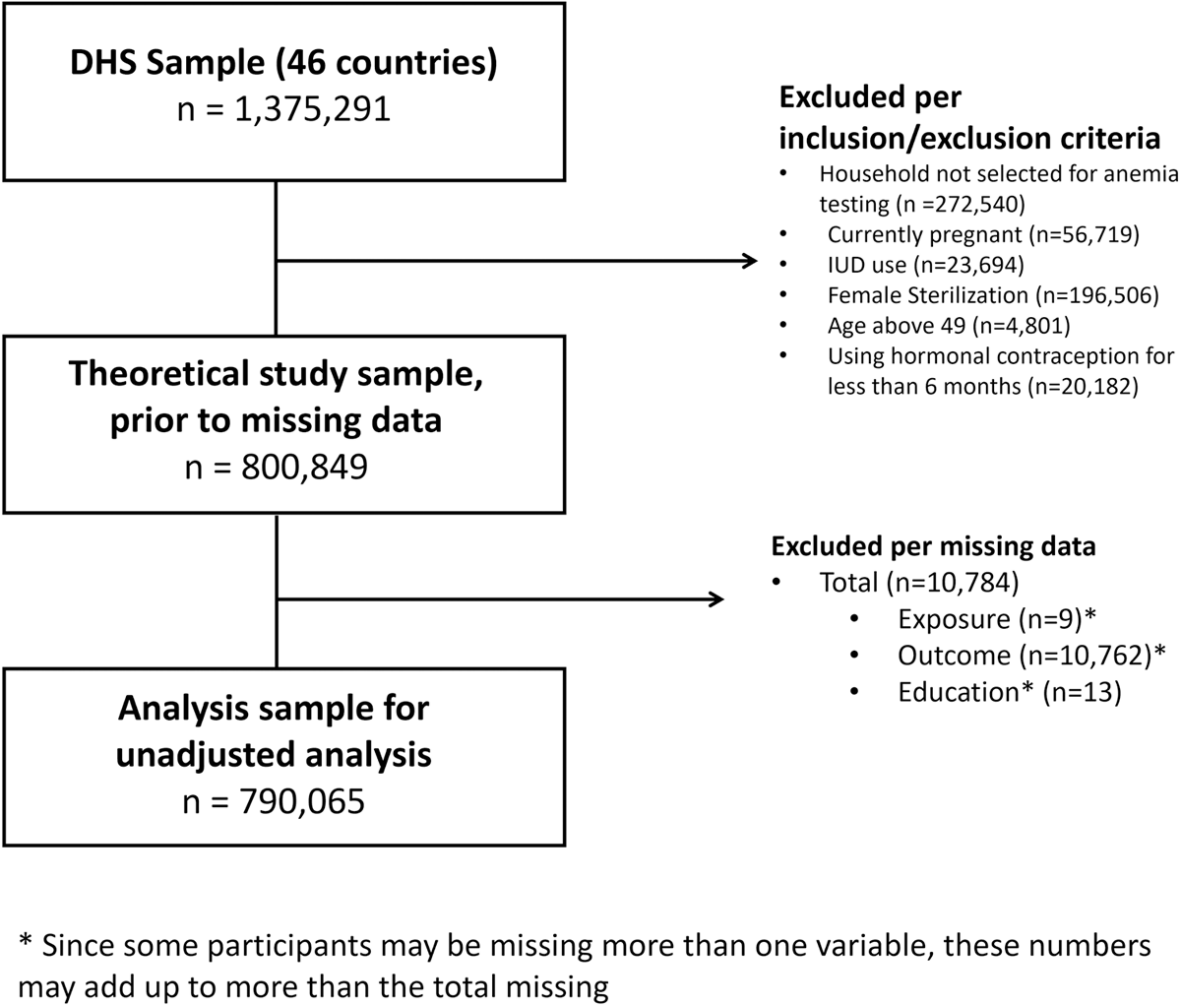
Flowchart detailing the merged DHS sample to the final analysis sample.

All 46 DHS combined included 1,375,291 women (Fig 2). Our theoretical analysis sample excluded households without anemia testing, and excluded women who were pregnant, used IUDs or female sterilization, or had been using hormonal contraceptives for less than six months. The final analysis sample included 790,065 women who were not missing exposure (n = 9), outcome (n = 10,762), or covariate data (n = 13 missing education).

### Anemia prevalence by country

**Fig 3:**
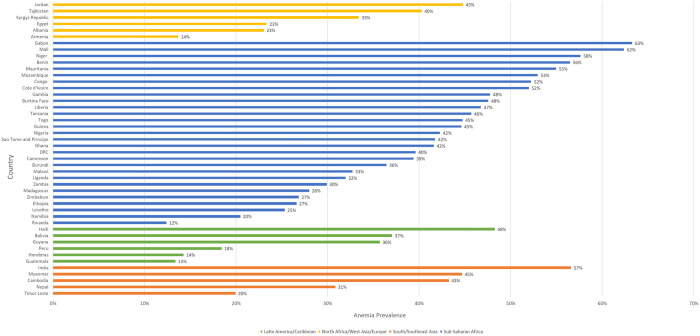
Weighted anemia prevalence (hemoglobin concentration below 12 g/dL) among non-pregnant women of reproductive age (15-49 years), by region and country, in 46 DHS conducted between 2008 and 2022.

Anemia prevalence ranged from 12.4% in Rwanda to 63.3% in Gabon, both countries in sub-Saharan Africa (Fig 3). Anemia prevalence ranged from 13.3% to 48.3% in the Latin America/Caribbean region (Guatemala and Haiti, respectively), from 13.7% to 44.8% in the North Africa/West Asia/Europe region (Armenia and Jordan, respectively), and from 19.9% to 56.6% in the South/Southeast Asia region (Timor Leste and India, respectively). Half of countries had an anemia prevalence over 40%, only six of which were outside of sub-Saharan Africa. Nine countries (i.e., Cote d’Ivoire, Congo, Mozambique, Mauritania, Benin, India, Niger, Mali, and Gabon) had an anemia prevalence over 50%.

### Odds of anemia with hormonal contraceptive use by country

**Fig 4:**
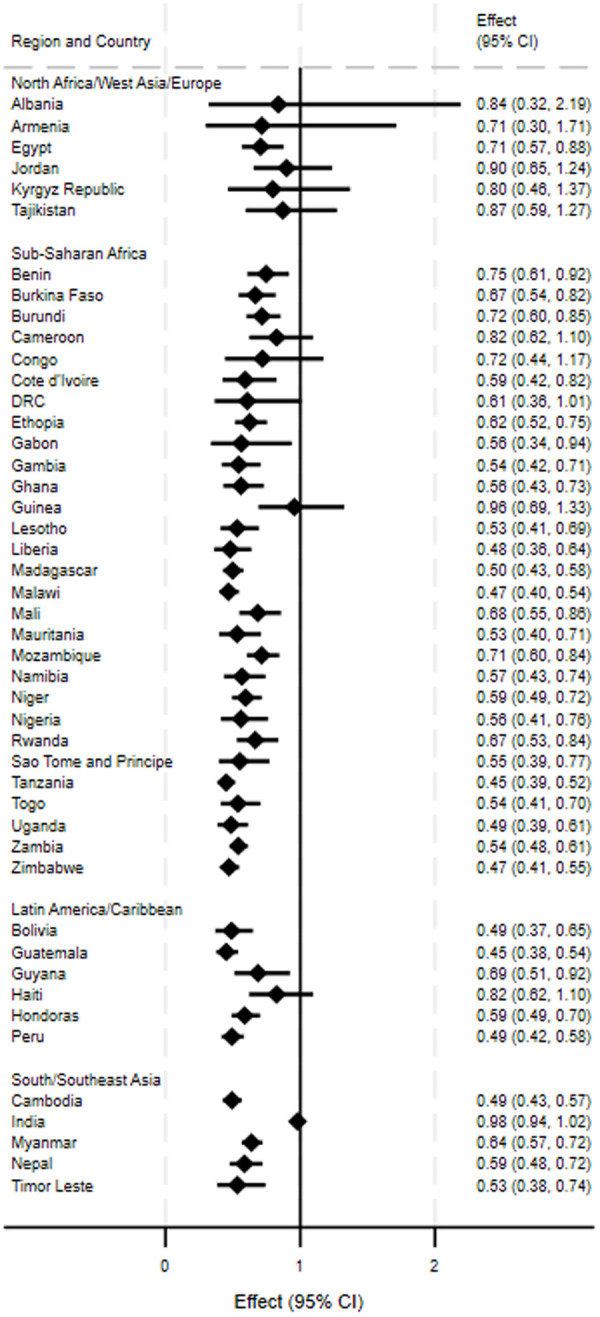
Odds ratios and 95% confidence intervals for anemia in non-pregnant women of reproductive age (15-49 years) using hormonal contraceptives for at least six months compared to nonusers of hormonal contraception adjusted for age, wealth, education, and rural residence, by region and country, across 46 DHS conducted between 2008 and 2022.

In each of the 46 countries, we compared odds of anemia in women using hormonal contraception (users) to those not using hormonal contraception (nonusers), controlling for age, wealth, education, and rurality (Fig 4). Overall, adjusted odds ratios (AOR) for anemia ranged from 0.45 in Tanzania (95% confidence interval: 0.39–0.52) and Guatemala (0.38–0.54) to 0.98 in India (0.94–1.02), indicating that users of hormonal contraception had lower odds of being anemic than non-users. In the sub-Saharan Africa region, AORs ranged from 0.45 in Tanzania to 0.96 in Guinea (0.69–1.33); in the Latin America/Caribbean region, AORs ranged from 0.45 in Guatemala to 0.82 in Haiti (0.62–1.10); in the North Africa/West Asia/Europe region, AORs ranged from 0.71 in Egypt (0.57–0.88) to 0.90 in Jordan (0.65–1.24); and in the South/Southeast Asia region from 0.49 in Cambodia (0.43–0.57) to 0.98 in India (0.94–1.02). While the difference between the odds ratios was lowest for the North Africa/West Asia/Europe region, this region also had estimates with low precision and without statistical significance. The odds of anemia in 35 countries were statistically significantly lower for users compared to nonusers at an alpha of 0.05; only 11 countries were not significant, including three countries in the sub-Saharan Africa region (i.e., Guinea, Cameroon, and the Democratic Republic of the Congo), Haiti in the Latin America/Caribbean region, five of the six countries in the North Africa/West Asia/Europe region (i.e., Albania, Armenia, Jordan, the Kyrgyz Republic, and Tajikistan), and India in the South/Southeast Asia region. While the estimates of odds of anemia ranged in precision, nearly 85% (39 out of 46) had confidence limit ratios under 2; those seven countries with a confidence limit ratio above 2 ranged from Tajikistan (2.14) to Albania (6.85) and were either in the North Africa/West Asia/Europe or Sub-Saharan Africa regions.

### Sample characteristics

When we combined data from the 46 DHS, nearly half (48%) of the merged sample had anemia (Table 1). Only 9% of the merged sample were users of hormonal contraception for at least six months, meaning 91% were non-users of hormonal contraception. Among users, 36% were anemic, whereas half (49%) of non-users were anemic, and anemia was lower in women who used injectable contraception and the contraceptive implant compared to those who used the pill. In this bivariate comparison, anemia varied somewhat by age, decreased as wealth and education increased, and was higher among those living in rural areas compared to urban areas. As mentioned above, India alone had a significant impact on these results and was therefore excluded from the moderation analysis. In the moderation analysis, we included those who had used contraception for any length of time. In this sample, 18% were using hormonal contraception, and 25% of hormonal contraceptive users were anemic. For a comparison of anemia by sociodemographic characteristics among all 46 countries, India alone, and 45 countries without India see S2 Table.

**Table 1:** Anemia by sociodemographic characteristics among non-pregnant women of reproductive age (15-49 years) across 46 or 45 DHS conducted between 2008 and 2022.

	Merged Analysis of 46 Countries		Moderation Analysis of 45^*^ Countries		
	Percent	Percent Anemic		Percent	Percent Anemic
Hormonal Contraceptive Use^†^					
Yes	9.0%	35.7%		17.5%	24.6%
Oral Contraceptive Pill	4.6%	46.8%		5.1%	26.5%
Injectable Contraception	3.3%	23.5%		9.4%	22.7%
Contraceptive Implant	1.1%	25.7%		3.0%	27.0%
No	91.0%	49.3%		82.5%	37.5%
Oral Contraceptive Pill					
6-12 months	0.7%	42.1%		0.8%	24.7%
13-24 months	0.1%	45.8%		0.9%	28.2%
25 months or more	3.0%	48.2%		1.9%	24.3%
Injectable contraception					
6-12 months	0.8%	26.3%		1.8%	23.9%
13-24 months	0.9%	22.8%		1.9%	20.8%
25 months or more	1.6%	22.3%		3.2%	19.5%
Contraceptive implant					
6-12 months	0.3%	25.7%		0.6%	25.7%
13-24 months	0.3%	24.4%		0.8%	24.4%
25 months or more	0.5%	26.6%		1.1%	26.6%
Age					
15-19	23.7%	49.9%		21.4%	35.3%
20-24	19.4%	48.8%		17.7%	34.5%
25-29	16.4%	47.5%		16.1%	34.0%
30-34	12.7%	46.4%		14.0%	34.6%
35-39	10.8%	47.3%		12.0%	36.1%
40-44	8.8%	47.1%		10.0%	37.1%
45-49	8.3%	47.1%		8.9%	36.1%
Parity					
0	39.3%	48.6%		31.1%	33.6%
1	16.3%	48.4%		15.2%	33.6%
2	17.3%	49.1%		15.3%	32.9%
3	10.4%	47.5%		11.8%	34.7%
4+	16.8%	46.0%		26.6%	39.5%
Education					
None	32.4%	49.6%		40.1%	39.5%
Primary	45.0%	49.5%		38.9%	34.0%
Secondary	7.5%	39.9%		11.3%	30.3%
Higher	15.2%	44.8%		9.7%	27.7%
Wealth					
Poorest	18.5%	54.1%		17.4%	38.7%
Poorer	19.5%	50.3%		18.6%	36.0%
Middle	19.7%	48.3%		19.7%	35.3%
Richer	20.6%	45.9%		21.2%	34.1%
Richest	21.6%	42.9%		23.0%	32.8%
Residence					
Urban	37.3%	44.2%		43.3%	34.0%
Rural	62.8%	50.4%		56.7%	36.1%
				
Total		47.9%			35.3%
N	790,065	378,232		342,479	120,733

Percentages are weighted using the provided DHS sampling weights.

* These columns exclude the India 2019/2021 DHS. Given the large sample size of India and the relatively higher prevalence of anemia in India, the percentages in the first column are greatly impacted by the inclusion of India in the sample. For a table comparing sociodemographic characteristics of the sample in all 46 countries, India alone, and 45 countries without India, see S2 Table.

† Users of hormonal contraceptives includes those who are using the contraceptive implant, oral contraceptive pill, and injectable contraception **for at least 6 months in the sample for the merged analysis**, and **any length of time for the sample in the moderation analysis**. Non-users of hormonal contraceptives includes those who use traditional methods (defined by the DHS as periodic abstinence, withdrawal, or country-specific methods, and folk methods), and modern methods that do not contain hormones (male sterilization, male or female condoms, foam/jelly, diaphragms, lactational amenorrhea method (LAM) and standard days method). Users of female sterilization and the IUD were excluded from the analytic sample.

### Merged analysis

In our regression model of the merged dataset, we found users of hormonal contraception for at least six months had 0.55 times the odds of having anemia compared to non-users when controlling for age, educational attainment, wealth, and rurality (Table 2). The 95% confidence interval for this odds ratio is 0.39 to 0.79, which indicates that these data are relatively compatible with a population level adjusted odds ratio between 0.39 and 0.79, conditional on age, educational attainment, wealth, and rurality, at an alpha of 0.05.

**Table 2:** Adjusted odds of anemia among non-pregnant women of reproductive age (15-49 years) by contraceptive and sociodemographic characteristics across 46 DHS between 2008 and 2022.

Variable	Odds Ratio	95% CI	p-value
Hormonal contraceptive use			
No (ref)	–	–	–
Yes	0.55	0.39-0.79	0.002
Wealth			
Poorest (ref)	–	–	–
Poorer	0.87	0.82-0.92	<0.001
Middle	0.82	0.73-0.91	0.001
Richer	0.76	0.64-0.91	0.004
Richest	0.70	0.55-0.91	0.008
Age			
15-19 (ref)	–	–	–
20-24	1.02	0.94-1.14	0.558
25-29	1.00	0.92-1.10	0.958
30-34	0.97	0.88-1.07	0.518
35-39	0.99	0.89-1.11	0.897
40-44	0.96	0.85-1.09	0.498
45-49	0.93	0.84-1.02	0.131
Education			
None (ref)	–	–	–
Primary	1.05	0.92-1.20	0.471
Secondary	0.74	0.57-0.96	0.023
Higher	0.93	0.75-1.16	0.501
Residence			
Urban (ref)	–	–	–
Rural	1.11	0.96-1.29	0.165
N	790,065		

### Moderation analysis

**Fig 5:**
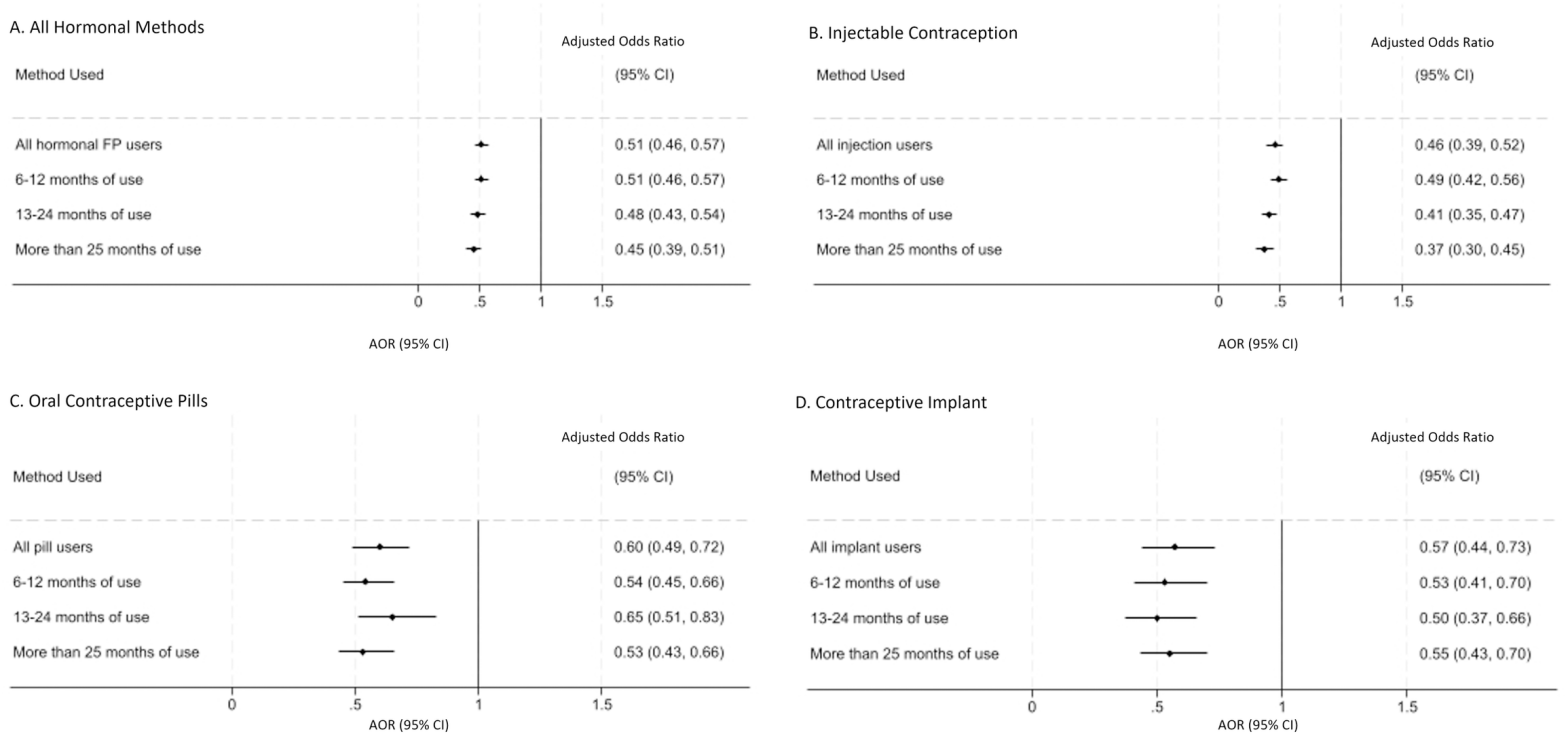
Odds ratios and 95% confidence intervals for anemia in non-pregnant women of reproductive age (15-49 years) using hormonal contraceptives compared to nonusers of hormonal contraception adjusted for age, wealth, education, and rural residence, stratified by method type and length of use, in 45 DHS conducted between 2008 and 2022.

To examine moderation effects by method type and duration of use, we only included 45 countries in the merged analysis. We decided to exclude the India DHS due to the results of our sensitivity analyses, which found a unique moderation effect by method type unseen in other countries (see Supplementary Material S1 Appendix for a detailed explanation of this sensitivity analysis and an exploration of the moderation analysis with all countries included). In the remaining 45 countries (n = 334,907), we found moderation effects by method type but not duration of use. Overall, those women using hormonal contraception for any length of time had 0.51 times the odds (0.46–0.57) of anemia compared to non-users of hormonal contraception, which decreased further to 0.45 times the odds (0.39–0.51) for those women using hormonal contraception for more than two years (Fig 5, panel A). The effect was strongest for users of the injectable contraception, with odds of anemia ranging from 0.46 (0.39–0.52) to 0.37 (0.30–0.45), depending on duration of use (Fig 5, panel B). The effect was more moderate for women using oral contraceptive pills, for whom the odds of anemia compared to non-users ranged from 0.6 (0.49–0.72) with any duration of use to 0.53 (0.43–0.66) for use of more than 2 years (Fig 5, panel C), and users of the contraceptive implant, where the effect ranged from 0.57 (0.44–0.73) with any duration of use to 0.55 (0.43–0.70) with more than two years of use (Fig 5, panel D).

### Analysis of anemia testing outcome misclassification

When we examined the effect of outcome misclassification by the HemoCue anemia testing system by varying the test specificity (i.e., ability to correctly identify those without anemia) and sensitivity (i.e., ability to correctly identify those with anemia), each between 100% and 75%, our crude unweighted odds ratio ranged from 0.29 to 0.57 (See S1 Table for details). If specificity stayed high, changes in sensitivity did not greatly affect our results; however, changes in specificity quickly changed the estimates. Regardless, we found our results are robust to high variation in sensitivity and specificity given we still found lower odds of testing positive for anemia among users of hormonal contraception compared to non-users in this analysis.

## Discussion

This analysis adds to a growing body of work examining the relationship between contraceptive use and anemia in LMICs. Our study differs from previous research in several key ways. First, we examined the impact of oral contraceptive pills, contraceptive implants, and injectable contraception, unlike many previous studies that have only focused on use of oral contraceptive pills [29,40] or use of hormonal IUDs [24–26]. Second, we did not limit our analysis to specific demographic populations, countries, or regions; instead, we included women of reproductive age in multiple LMICs across four global regions in our comparative analysis. We found women using hormonal contraception had nearly half the odds of having anemia of women who were not using hormonal contraception, controlling for age, wealth, educational attainment, and area of residence. These findings are consistent with other similar studies of the relationship between anemia and contraceptive use and support the growing evidence that hormonal contraceptive use has a causative impact on anemia [28,29,31]. Especially in the Latin America/Caribbean, South/Southeast Asia, and sub-Saharan African regions, our estimates indicated hormonal contraceptive use is associated with lower odds of being anemic.

Third, although other analyses have also used DHS data to examine multiple hormonal contraceptive methods in aggregate, we conducted novel stratified analyses by both method type and length of use to examine their moderation effect and addressed concerns about the accuracy of the anemia testing system used by the DHS by assessing if our results are robust to measurement error [28,31,34,35]. Building upon the previous research that focused on oral contraceptive and IUD use, our moderation analysis found that the protective effect of hormonal contraceptive use is greater for injectable contraception compared to oral contraceptive pills at 13–24 months. This is likely due to level of user involvement and the differential impacts of these forms of contraception on the menstrual cycle. Injectable contraception is more likely than contraceptive implants to completely pause bleeding, especially with extended use, and oral contraceptive pills include combined hormonal pills, which are designed to mimic monthly menstrual bleeding with withdrawal bleeding due to the 4–7 days of placebo pills [19–21]. For oral contraceptive pills, users must take them daily, whereas injectable contraception requires less user involvement, with one visit every three months. This lack of effect by duration of use differs from what previous studies on the relationship between oral contraceptive use and anemia have found, notably a similar DHS analysis including 12 countries [29]. Additionally, there are fewer implant users in our sample, which may make the confidence interval for our estimate less precise. We did not find significant effects for duration of use within methods; while the point estimate was lower with longer duration of use for most methods, there was still overlap in confidence intervals when comparing longer and shorter durations of use.

Our use of DHS data for this analysis has both strengths and limitations. Data collected in a standardized way across countries allows us to examine broader trends across countries and regions. On the other hand, we lose the nuance of doing a more in-depth analysis of the unique context of one country. The DHS is not done in every country; this and our exclusion criteria limits our ability to make strong regional comparisons. The rigorous complex sampling design mitigates concerns about selection bias and the quality of data collection with generally low levels of missing data (e.g., we were only missing data on 10 individuals for the exposure in a sample of over 1 million women). Still, each country and regions within countries have unique characteristics, and DHS data, which are designed to be comparative across countries, limit in our ability to control for country-specific confounders. Although the DHS wealth index can account for some measures of social inequities, we are still unable to control for country-specific factors that could greatly affect anemia, including nutritional status or infectious disease. The cross-sectional nature of the DHS also limits our ability to draw causal inferences. Furthermore, because the DHS does not distinguish between the copper and hormonal IUD, we had to exclude all IUD users from our analysis given their very different impacts on the menstrual cycle, despite promising evidence of the impact of the hormonal IUD on anemia [24,26,27]. While some countries opt in to collecting data about brands of pills or condoms, the DHS does not collect data about brands or formulations of implants and injections. For injections specifically, the different menstrual bleeding patterns seen with progestin-only injectables compared to combined injectable contraception (CIC) should be taken into consideration in countries where CIC use is high [53]. Additionally, the DHS does not collect more granular data on variables like menstruation that would have been helpful to further explore possible mediating pathways between hormonal contraceptive use and anemia status.

Despite these limitations, our findings strongly suggest hormonal contraceptives may be a useful way to address anemia in women of reproductive age, and this impact may be stronger for longer acting methods that require low user involvement and/or completely pause bleeding. Our moderation analysis provides evidence that contraceptive implant and injectable contraceptive use may result in a protective effect on anemia. Our analysis for misclassification of the outcome, which is a significant concern with the HemoCue+ systems, strengthen this evidence. Even if we assumed that the sensitivity and specificity were as low as 75%, we still found hormonal contraceptive users had lower odds of having anemia compared to nonusers.

Continued research is necessary to establish a causal link between hormonal contraceptive use and anemia reduction. For example, the LISA randomized controlled trial [23] is currently exploring associations between use of hormonal IUDs and anemia status. Still, this paper adds to the growing evidence that anemia prevention is a notable non-contraceptive benefit of certain types of hormonal contraception, including contraceptive implants and injectable contraception [28–31]. This link between anemia and contraceptive use is a promising point for intervention as we expand options for treatment of and prevention of anemia, but research and translation of that research into practice in these areas remain largely siloed between those studying nutrition and family planning. Interdisciplinary work between these related fields is greatly needed in order to begin to understand the nutrition, maternal and child health, and family planning policy implications of these findings and ensure that the impact of menstrual blood loss on anemia is prioritized and addressed in detail in international anemia guidelines that currently include very little information about this potential treatment or prevention strategy [54]. Hormonal contraception should be considered one important part of a multipronged approach to preventing and treating anemia in women of reproductive age who are using or considering contraception, especially in contexts where significant improvements are needed to address other causes of anemia, such as food quality and scarcity, poverty, and infectious diseases. However, the potential for anemia treatment or prevention should not be a reason that women with anemia are disproportionately steered towards hormonal contraception. Instead, the anemia reducing benefits of contraception are a promising piece of wider efforts to address anemia, and this information provides women with a more comprehensive picture of non-contraceptive benefits to inform their contraceptive decision making as they exercise their reproductive autonomy.

## Supporting information

S1 File Supplementary material for “Estimating the Effect of Hormonal Contraceptive Use on Anemia: A Cross-sectional Comparative Analysis of 46 Demographic and Health Surveys”. (DOCX)
