# Supplementary material for: Estimating the effect of hormonal contraceptive use on anemia: A cross-sectional comparative analysis of 46 Demographic and Health Surveys
Source: PLoS One. 2025 Jul 16;20(7):e0327083. doi: 10.1371/journal.pone.0327083 (PMC12266412; doi:10.1371/journal.pone.0327083)
Supplement: S1 File — (DOCX) [file pone.0327083.s001.docx]

For: Estimating the Effect of Hormonal Contraceptive Use on Anemia: A Cross-sectional Comparative Analysis of 46 Demographic and Health Surveys

Supplementary Material

**S1 Fig. Directed acyclic graph (DAG) illustrating relationship between hormonal contraceptive use and anemia.**

**
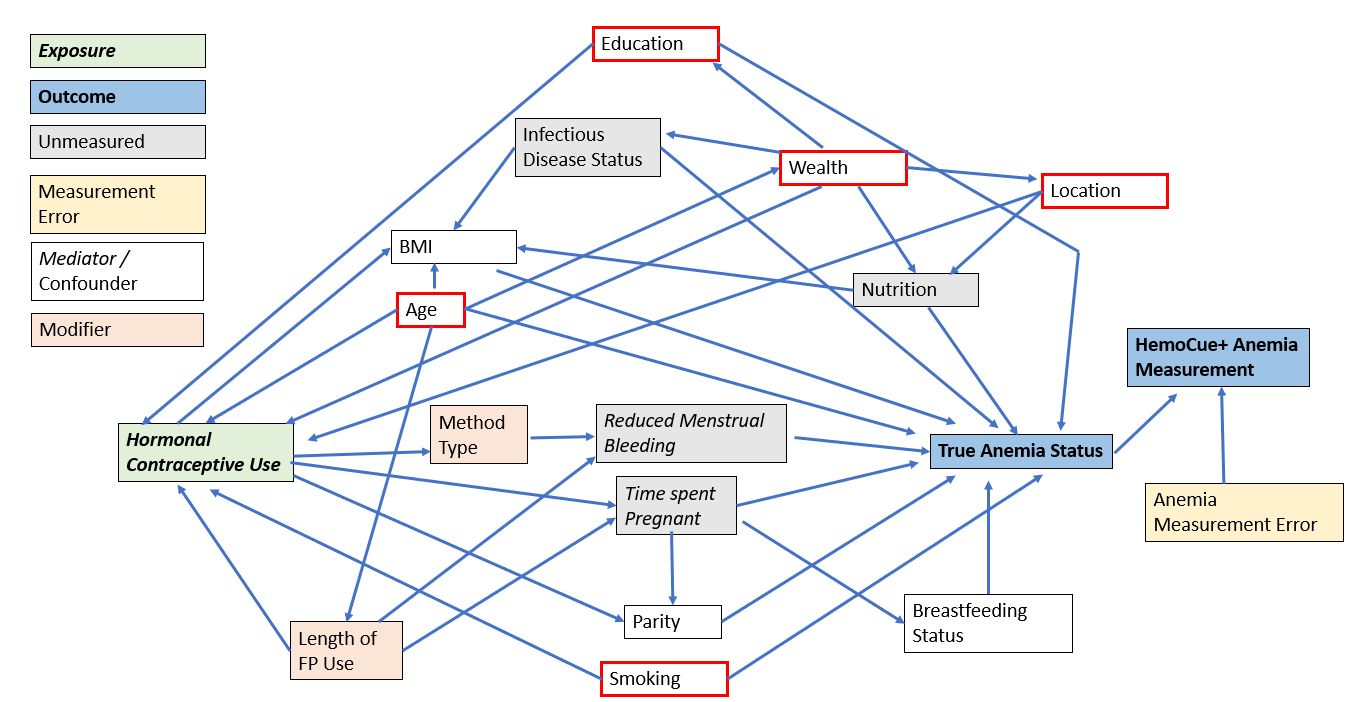
**

*Controlling or adjusting for the variables outlined in red closes ‘back-door’ paths, which allows us to estimate the total effect of hormonal contraceptive use on anemia status.*

| **S1 Table. Crude, unweighted odds ratio of testing positive for anemia when varying sensitivity and specificity of HemoCue+ test, comparing users of hormonal contraception to non-users among non-pregnant women of reproductive age (15-49 years) in 46 DHS conducted between 2008 and 2022 (n=790,065).** | | | | | | | | | | | |
| --- | --- | --- | --- | --- | --- | --- | --- | --- | --- | --- | --- |
|  | **Specificity** | | |  | |  | |  | |  | |
| **Sensitivity** | | **1** | **0.95** | | **0.9** | | **0.85** | | **0.8** | | **0.75** |
| **1** | | 0.57 | 0.55 | | 0.52 | | 0.48 | | 0.42 | | 0.35 |
| **0.95** | | 0.56 | 0.54 | | 0.51 | | 0.47 | | 0.41 | | 0.34 |
| **0.9** | | 0.55 | 0.52 | | 0.49 | | 0.45 | | 0.4 | | 0.33 |
| **0.85** | | 0.53 | 0.5 | | 0.48 | | 0.44 | | 0.39 | | 0.32 |
| **0.8** | | 0.5 | 0.48 | | 0.46 | | 0.42 | | 0.37 | | 0.31 |
| **0.75** | | 0.48 | 0.46 | | 0.43 | | 0.4 | | 0.35 | | 0.29 |

**Sensitivity Testing of Moderation Analysis**

For the moderation analysis, we first stratified by method type and duration of use in the complete merged sample of 46 countries. Additionally, we performed sensitivity analyses to investigate if these results were being biased by countries with large sample sizes or those with high overall rates of anemia with low hormonal contraceptive use. While we conducted sensitivity analyses for three countries (India, Gabon, and Nigeria), here we present the results of the moderation analysis with and without India, as this had the largest impact on our results.

We decided to explore the impact of removing India from our analysis sample due to substantial differences between our sample of women of reproductive age in India and the other 45 countries. India was chosen for the sensitivity analysis because it comprised over half of our sample (58.6%), had a low proportion of hormonal contraceptive users and high prevalence of anemia, both of which could have impacted the results (Table S2). The 2019-2021 India DHS reported a high overall prevalence of anemia at 56.6% of the total sample – compared to 35.3% of the merged sample of the 45 other countries. Additionally, India reported low hormonal contraceptive use (5.6%) and similar prevalence of anemia between hormonal contraceptive users (58.1%) and non-users (57.4%). This contrasts with the merged sample of the 45 other countries, which had higher hormonal contraceptive use (17.5%) and a difference in anemia status between hormonal contraceptive users and non-users (24.6% among users compared to 37.5% in non-users). Notably, India has very few users of contraceptive implants and injectable contraception, which could have impacted the moderation analysis results.

**S2 Table. Anemia status by sociodemographic characteristics among non-pregnant women of reproductive age (15-49 years) compared between 46 DHS conducted between 2008 and 2022, the 2019-2021 India DHS, and 45 DHS conducted between 2008 and 2022, without India.**

|  |  |  |  |  |  |  |  |  |
| --- | --- | --- | --- | --- | --- | --- | --- | --- |
|  | Merged Sample of 46 countries | |  | India, 2019-2021 | |  | Merged sample of 45 countries (without India) | |
|  | Percent | Percent Anemic |  | Percent | Percent Anemic |  | Percent | Percent Anemic |
| Hormonal Contraceptive Use † |  |  |  |  |  |  |  |  |
| **Yes** | 9.0% | 35.7% |  | 5.6% | 58.1% |  | 17.5% | 24.6 % |
| Oral Contraceptive Pill | 4.6% | 46.8% |  | 5.1% | 58.8% |  | 5.1% | 26.5% |
| Injectable Contraception | 3.3% | 23.5% |  | 0.5% | 50.9% |  | 9.4% | 22.7% |
| Contraceptive Implant | 1.1% | 25.7% |  | - | - |  | 3.0% | 27.0% |
| **No** | 91.0% | 49.3% |  | 94.4% | 57.4% |  | 82.5% | 37.5% |
| Oral Contraceptive Pill |  |  |  |  |  |  |  |  |
| 6-12 months | 0.7% | 42.1% |  | 0.6% | 60.0% |  | 0.8% | 24.7% |
| 13-24 months | 0.1% | 45.8% |  | 0.8% | 61.0% |  | 0.9% | 28.2% |
| 25 months or more | 3.0% | 48.2% |  | 3.6% | 58.1% |  | 1.9% | 24.3% |
| Injectable contraception |  |  |  |  |  |  |  |  |
| 6-12 months | 0.8% | 26.3% |  | 0.1% | 56.2% |  | 1.8% | 23.9% |
| 13-24 months | 0.9% | 22.8% |  | 0.1% | 50.8% |  | 1.9% | 20.8% |
| 25 months or more | 1.6% | 22.3% |  | 0.3% | 48.6% |  | 3.2% | 19.5% |
| Contraceptive implant |  |  |  |  |  |  |  |  |
| 6-12 months | 0.3% | 25.7% |  | - | - |  | 0.6% | 25.7% |
| 13-24 months | 0.3% | 24.4% |  | - | - |  | 0.8% | 24.4% |
| 25 months or more | 0.5% | 26.6% |  | - | - |  | 1.1% | 26.6% |
| Age |  |  |  |  |  |  |  |  |
| 15-19 | 23.7% | 49.9% |  | 25.1% | 59.1% |  | 21.4% | 35.3% |
| 20-24 | 19.4% | 48.8% |  | 21.0% | 57.2% |  | 17.7% | 34.5% |
| 25-29 | 16.4% | 47.5% |  | 16.8% | 56.6% |  | 16.1% | 34.0% |
| 30-34 | 12.7% | 46.4% |  | 11.8% | 56.2% |  | 14.0% | 34.6% |
| 35-39 | 10.8% | 47.3% |  | 9.9% | 57.1% |  | 12.0% | 36.1% |
| 40-44 | 8.8% | 47.1% |  | 7.7% | 56.8% |  | 10.0% | 37.1% |
| 45-49 | 8.3% | 47.1% |  | 7.7% | 56.9% |  | 8.9% | 36.1% |
| Parity |  |  |  |  |  |  |  |  |
| 0 | 39.3% | 48.6% |  | 44.4% | 56.6% |  | 31.1% | 33.6% |
| 1 | 16.3% | 48.4% |  | 17.4% | 57.3% |  | 15.2% | 33.6% |
| 2 | 17.3% | 49.1% |  | 19.0% | 58.3% |  | 15.3% | 32.9% |
| 3 | 10.4% | 47.5% |  | 9.5% | 58.6% |  | 11.8% | 34.7% |
| 4+ | 16.8% | 46.0% |  | 9.7% | 58.4% |  | 26.6% | 39.5% |
| Education |  |  |  |  |  |  |  |  |
| None | 32.4% | 49.6% |  | 26.8% | 60.3% |  | 40.1% | 39.5% |
| Primary | 45.0% | 49.5% |  | 49.6% | 58.2% |  | 38.9% | 34.0% |
| Secondary | 7.5% | 39.9% |  | 4.6% | 57.1% |  | 11.3% | 30.3% |
| Higher | 15.2% | 44.8% |  | 19.1% | 51.3% |  | 9.7% | 27.7% |
| Wealth |  |  |  |  |  |  |  |  |
| Poorest | 18.5% | 54.1% |  | 19.3% | 64.0% |  | 17.4% | 38.7% |
| Poorer | 19.5% | 50.3% |  | 20.2% | 59.8% |  | 18.6% | 36.0% |
| Middle | 19.7% | 48.3% |  | 19.7% | 57.6% |  | 19.7% | 35.3% |
| Richer | 20.6% | 45.9% |  | 20.2% | 54.7% |  | 21.2% | 34.1% |
| Richest | 21.6% | 42.9% |  | 20.5% | 51.2% |  | 23.0% | 32.8% |
| Residence |  |  |  |  |  |  |  |  |
| Urban | 37.3% | 44.2% |  | 32.6% | 54.1% |  | 43.3% | 34.0% |
| Rural | 62.8% | 50.4% |  | 67.4% | 59.0% |  | 56.7% | 36.1% |
|  |  |  |  |  |  |  |  |  |
| Total |  | 47.9% |  |  | 56.6% |  |  | 35.3% |
| N | 790,065 | 378,232 |  | 463,262 | 262,019 |  | 342,479 | 120,733 |

In the complete merged sample of 46 countries, when we stratified results by method type, we found women using injectable contraception had 0.33 times the odds (95% confidence interval [CI]: 0.27-0.41) of having anemia, and women who used the contraceptive implant had 0.37 times the odds (0.24-0.58) of having anemia compared to non-users of hormonal contraceptives (Figure S2, panels B and D), both when controlling for age, wealth, education, and rurality. Those who used the oral contraceptive pill were no more or less likely to have anemia compared with non-users of a hormonal method (adjusted odds ratio (AOR)=0.85, 95% CI: 0.64-1.13) (Figure S2, panel C). When we examined results by duration of use, the odds of anemia among users of hormonal contraception were similar for all pill use, and pill use of at least 6 months, 1 year, and 2 years (AOR=0.55, 0.39-0.75; AOR=0.48, 0.38-0.60; AOR=0488, 0.36-0.65; and AOR=0.62, 0.42-0.91, respectively; Figure S2, panel A). This was also true for injectable contraception, the contraceptive implant, and oral contraceptive pills, when further stratified by method (Figure S2, panels B-D). We conducted a sensitivity analysis to determine if these results were impacted by removing those with recent (within the last 6 months) discontinuation of a hormonal contraceptive method; we found changes to the effect estimates and 95% CIs were less than 0.01 in every case except for any duration of pill use, where there was a change of 0.03 (AOR 0.88, 0.68-1.14, not shown).

**S2 Fig. Odds ratios and 95% confidence intervals for anemia in non-pregnant women of reproductive age (15-49 years) using hormonal contraceptives compared to nonusers of hormonal contraception adjusted for age, wealth, education, and rural residence, stratified by method type and length of use, in 46 DHS conducted between 2008 and 2022.**


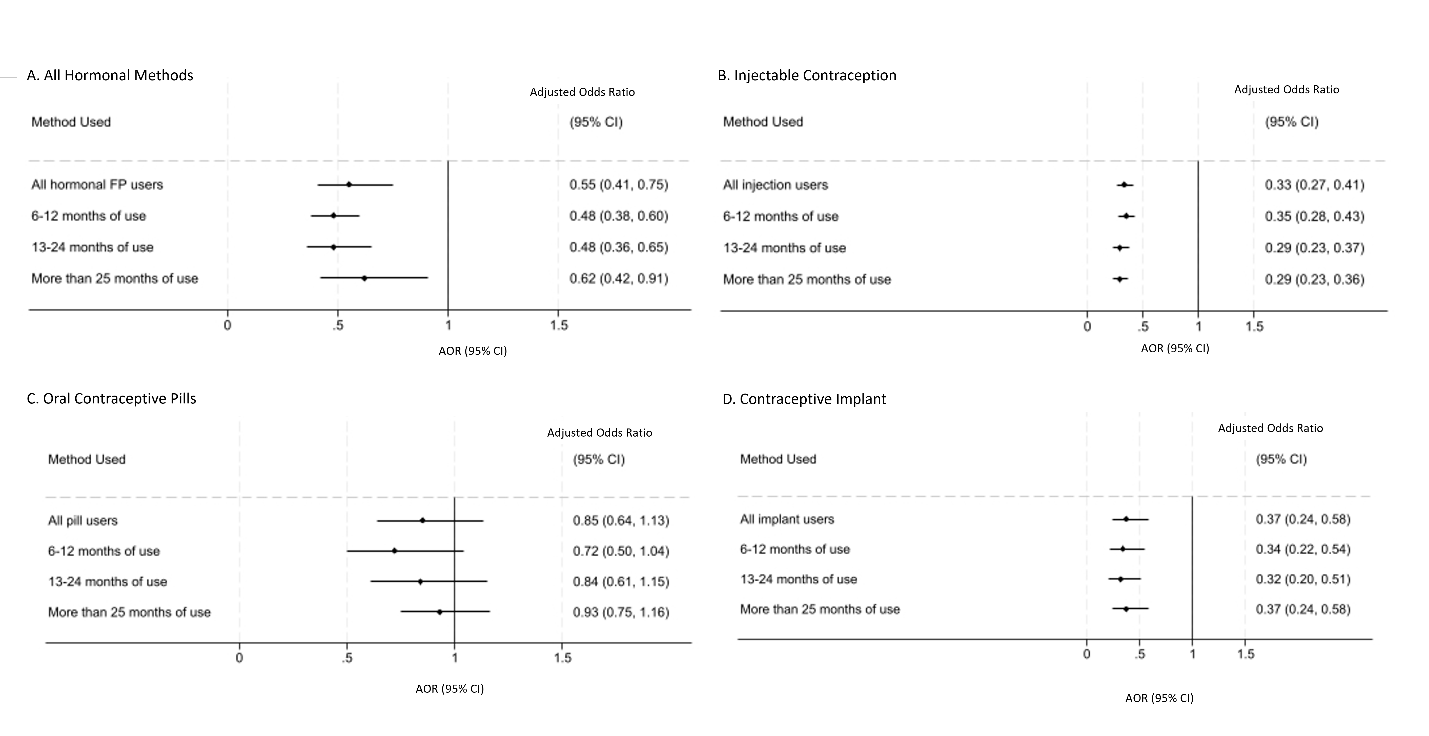


When we look at India alone, we see no effect of using hormonal contraception or using the oral contraceptive pill (Figure S3, panels A and C). However, use of injectable contraception is significant and does show a mild moderation effect with duration of use with odds of anemia between users of injectable contraception for any length of time being 0.78 (95% CI: 0.71-0.86) times that of non-users of hormonal contraception, and for odds of anemia among users of injectable contraception of at least two years being 0.70 (95% CI: 0.60-0.81) times that of non-users (Figure S3, panel B). Notably, India does not have enough users of contraceptive implants to draw any conclusions about the impact of contraceptive implant use on anemia status.

**S3 Fig. Odds ratios and 95% confidence intervals for anemia in non-pregnant women of reproductive age (15-49 years) using hormonal contraceptives compared to nonusers of hormonal contraception adjusted for age, wealth, education, and rural residence, stratified by method type and length of use, in the 2019-2021 India DHS.**

**
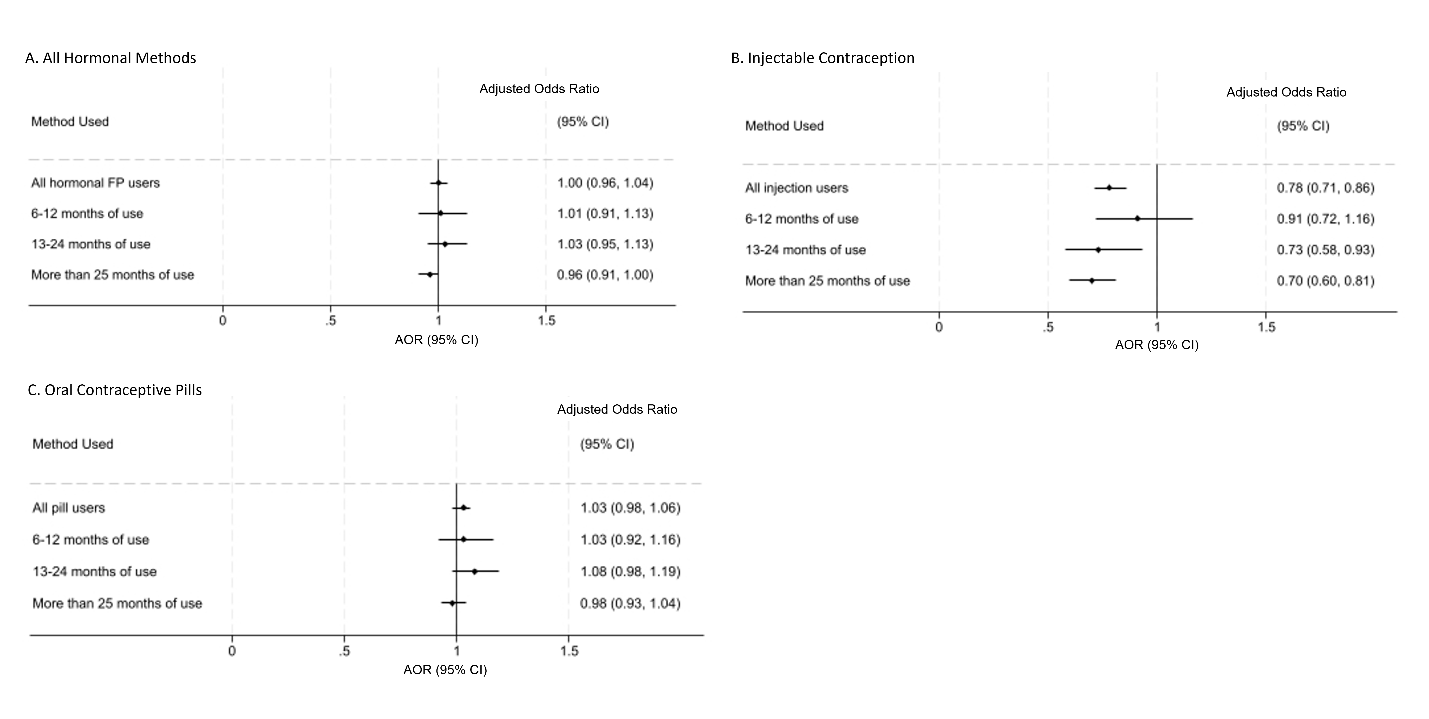
**

Based on these findings, the impact of hormonal contraceptive use on anemia status seems to be different in India than it is among the other 45 countries in our sample. This could be due to the high overall prevalence of anemia in India (56.6% compared to 35.5%), and the high proportion of moderate anemia (28.4%) in India compared to the rest of the sample (10.8%). Another potential explanation is the low prevalence of hormonal contraceptive use in India, and the higher usage of female sterilization and IUDs in India. We hypothesize that including India in the moderation analysis would unduly bias the results we present in the main analysis (Figure 5) to no longer be more widely representative, especially the results for oral contraceptive pill users and users of contraceptive implants. For example, as there are not many implant users in India, including India in that moderation analysis would add a large number of anemic individuals into the reference group (non-users of hormonal contraception), thus exaggerating the effect of contraceptive implant use on anemia status. Therefore, we present the more representative moderation analysis without India in the main paper to avoid this source of potential bias.
